# Correction: Water-induced formation of an alkali-ion dimer in cryptomelane nanorods

**DOI:** 10.1039/d0sc90126a

**Published:** 2020-07-02

**Authors:** Shaobo Cheng, Vidushi Sharma, Altug S. Poyraz, Lijun Wu, Xing Li, Amy C. Marschilok, Esther S. Takeuchi, Kenneth J. Takeuchi, Marivi Fernández-Serra, Yimei Zhu

**Affiliations:** Department of Condensed Matter Physics and Materials Science, Brookhaven National Laboratory Upton NY 11973 USA zhu@bnl.gov; Department of Physics and Astronomy, Stony Brook University Stony Brook NY 11794-3800 USA maria.fernandez-serra@stonybrook.edu; Institute for Advanced Computational Science, Stony Brook University Stony Brook NY 11794 USA; Energy Sciences Directorate, Brookhaven National Laboratory Upton NY 11973 USA; Department of Chemistry and Biochemistry, Kennesaw State University Kennesaw GA 30144 USA; School of Physics and Microelectronics, Zhengzhou University Daxue Road 75 Zhengzhou 450052 China; Department of Chemistry, Stony Brook University Stony Brook NY 11794 USA; Department of Materials Science and Chemical Engineering, Stony Brook University Stony Brook NY 11794 USA

## Abstract

Correction for ‘Water-induced formation of an alkali-ion dimer in cryptomelane nanorods’ by Shaobo Cheng *et al.*, *Chem. Sci.*, 2020, **11**, 4991–4998, DOI: 10.1039/D0SC01517B.

We regret that due to a conversion error all instances of the chemical formula K_2_^+^ appear incorrectly as K^2+^ in this article.

The Royal Society of Chemistry apologises for these errors and any consequent inconvenience to authors and readers.

